# What can ecosystem models tell us about the risk of eutrophication in the North Sea?

**DOI:** 10.1007/s10584-014-1071-x

**Published:** 2014-03-22

**Authors:** S. Saux Picart, J. I. Allen, M. Butenschön, Y. Artioli, L. de Mora, S. Wakelin, J. Holt

**Affiliations:** 1 Plymouth Marine Laboratory, Prospect Place, The Hoe, PL1 3DH Plymouth, UK; 2 National Oceanography Centre, 6 Brownlow Street, L3 5DA Liverpool, UK

## Abstract

Eutrophication is a process resulting from an increase in anthropogenic nutrient inputs from rivers and other sources, the consequences of which can include enhanced algal biomass, changes in plankton community composition and oxygen depletion near the seabed. Within the context of the Marine Strategy Framework Directive, indicators (and associated threshold) have been identified to assess the eutrophication status of an ecosystem. Large databases of observations (*in situ*) are required to properly assess the eutrophication status. Marine hydrodynamic/ecosystem models provide continuous fields of a wide range of ecosystem characteristics. Using such models in this context could help to overcome the lack of *in situ* data, and provide a powerful tool for ecosystem-based management and policy makers. Here we demonstrate a methodology that uses a combination of model outputs and *in situ* data to assess the risk of eutrophication in the coastal domain of the North Sea. The risk of eutrophication is computed for the past and present time as well as for different future scenarios. This allows us to assess both the current risk and its sensitivity to anthropogenic pressure and climate change. Model sensitivity studies suggest that the coastal waters of the North Sea may be more sensitive to anthropogenic rivers loads than climate change in the near future (to 2040).

## Introduction

Shelf seas are the most highly productive regions of the world ocean providing a diverse range of goods (e.g., food, renewable energy) and services (e.g., carbon, nutrient cycling, biodiversity and transport). Global climate change will lead to large scale changes in theirphysical conditions (i.e., circulation, stratification, temperature, and light) and consequently in their biogeochemistry (Steinacher et al. 2010). Simultaneously combinations of direct anthropogenic drivers (e.g., fishing, and eutrophication) will impact at both an organism and population level thereby influencing the biogeochemical cycles of carbon and nutrients. Many coastal regions are at risk from both eutrophication and climate change. Eutrophication is a process resulting from an increase in anthropogenic nutrient inputs from rivers and other sources; the consequences of which can include enhanced algal biomass, changes in plankton community composition and oxygen depletion near the seabed. All of these effects have been observed in the southern North Sea (e.g., Lenhart et al. 2010; Weston et al.^2008^). Regionally downscaled climate forced simulations of marine ecosystems indicate that increased stratification in the NE Atlantic leads to a reduced oceanic nutrient supply into the North Sea, potentially leading to a significant reduction in primary production by 2100 (Holt et al. 2012).

The Marine Strategy Framework Directive (MSFD, European Commission 2008) requires EC member states to develop strategies to achieve a healthy marine environment and make ecosystems more resilient to climate change in all European marine waters by 2020 at the latest. These strategies must contain a detailed assessment of the state of the environment, a definition of “Good Environmental Status” (GES) at regional level and the establishment of clear environmental targets and monitoring programmes.

Eutrophication is a key topic and in a policy context is defined as ‘the enrichment of water by nutrients causing accelerated growth of algae and other higher forms of life to produce an undesirable disturbance of the balance of organisms present in the water and the quality of the water concerned’(OSPAR 2003).

Various tools have been developed to quantitatively assess the eutrophication status of marine ecosystems. Uses and applications of such tools are numerous, for example the tools used by HELCOM (2006, 2009) for the Baltic Sea, and OSPAR (2005, 2008) for the North-East Atlantic. Many of them are based on indicators used in combination to provide a holistic view of the eutrophication status. These indicators include, among others, winter nitrate, summer chlorophyll, summer oxygen or winter nitrate-to-phosphate ratio, and have been chosen because they represent fundamental aspects of the system (chemical energy to drive growth, algal biomass, impacts of high biomass) and have thresholds which define the point at which harmful effects occur.

A range of coupled hydrodynamic ecosystem models have been used to assess the consequences of nutrient reduction on the eutrophication status of the North Sea (e.g., Lenhart 2001; Lacroix et al. 2007; Skogen and Mathisen 2009; Lenhart et al. 2010). However these models have not been used to evaluate the risk of eutrophication and its relative sensitivity to either changes in anthropogenic loadings or to climate change in future scenarios. In the present study, we designed a methodology to quantify the risk of eutrophication from model output in terms of percentage exceedance of thresholds. This quantification potentially gives more information than a widely used “above/below threshold” metric. The method is here developed for the North Sea coastal domain as a whole using selected indicators and associated threshold. Being able to quantify the risk of eutrophication from model outputs for a predefined ecosystem or region would give ecosystem-based management a considerable help. In fact this would add an operational (and possibly a forecasting) dimension to the monitoring of the risk of eutrophication.

## Methodology

### Indicators used for eutrophication risk assessment

Karydis (2009) and Ferreira et al. (2011) give a comprehensive review of indicators developed and used to assess eutrophication status. Ferreira et al. (2011) highlighted that evaluating confidence in the results should not be neglected and difficulties associated with spatial and temporal scales of the assessment should be addressed. McQuatters-Gollop et al. (2009) emphasise the importance of using a suite of indicators and assessing the eutrophication status on similar spatial scales if one is to consider different regions.

It is now widely accepted that assessment indicators of eutrophication are divided into three to four categories which includes causative factors, direct and indirect effects (and eventually other possible effects).

Amongst the variety of indicators commonly used to evaluate the eutrophication status, we chose a sub-sample for which a large number of measurements are available (see Section 2.2), and we define thresholds drawing on the Common Procedure (OSPAR 2005) as a basis to develop and demonstrate our methodology. The chosen indicators and their associated threshold are:

- Winter (November-February) nitrate concentration: threshold defined as above 20 *μ*M for coastal waters
- Spring-summer (March-September) chlorophyll concentration: threshold defined as above 15 *μ*g l^−1^ for coastal waters
- Summer oxygen (May-September) concentration: threshold defined as below 5 mg l^−1^ for coastal waters

Winter phosphate is also an indicator commonly used. We have tested it, but since it follows closely the behavior of the nitrate indicator, we have chosen not to include it here.

Eutrophication is a problem observed mainly in coastal or estuarine regions. We therefore chose to carry out our study in coastal waters of the North Sea (corresponding to region IV of the International Council for the Exploration of the Sea). The coastal domain is defined by using a salinity criteria (30.5 < Salinity < 34.5).

### Data, model and scenarios

For the purpose of this study we use the European Regional Seas Ecosystem Model (ERSEM, Baretta et al. 1995; Blackford et al. 2004) coupled with the Proudman Oceanographic Laboratory Coastal-Ocean Modelling System (POLCOMS, Holt and James 2001) hydrodynamic model. ERSEM is a lower-trophic level biogeochemical cycling model, explicitly resolving the Carbon, Nitrogen, Oxygen, Phosphorus, and Silicon cycles through both the benthic and pelagic ecosystems. The pelagic food web is composed of functional types (four phytoplankton, three zooplankton and one bacterium), while the benthic sub-model resolves bacteria, meio- and macrofauna.

The Atlantic Margin Model (AMM) domain, which includes the whole North Sea, was used. This domain has a horizontal resolution of 1/6° by 1/9° (approximately 12 × 12km) and use *s*-coordinates in the vertical with 40 layers. The time step is 20 minutes for ERSEM and 5 minutes for POLCOMS, but daily averaged outputs were used. A complete description of the POLCOMS-ERSEM configuration used is available in Holt et al. (2012).

Our assesment of the impact of changes in riverine nutrient loads on the eutrophication status is based on five sets of simulations:

- A hindcast (1965-2004) with atmospheric forcing from European Centre for Medium-Range Weather Forecasts (ECMWF) ERA-40 renanalysis until 2002 and ECMWF operational analysis after Wakelin et al. (2012), used for validation of the methodology against *in situ* data.
- A present day (PD) simulation (1981-2000) forced by the Institut Pierre Simon Laplace Climate Model (IPSL-CM4, Marti et al. 2010). This is used to define the parameters of the methodology to be applied in the future projections and as baseline to assess impact of climate change.
- Three future projections to assess the impact of changes in riverine nutrient loads:
  - A reference future projection forced by the IPSL simulations under the Intergovernmental Panel on Climate Change Assessment Report (IPCC-AR4) A1B scenario for the years 2030-40 with identical riverine inputs with respect to the present day run.
  - A World Market (WM) scenario based on the same A1B projection, but with riverine inputs reflecting rapid economic growth and limited environmental policies with a relative decline in agriculture and manufacturing compared to the service sector. In the simulations this is translated to a +50 % increase in river nitrogen and constant phosphate (with respect to the present day run) based on the results of the European Lifestyles and Marine Ecosystems (ELME) project (Langmead et al. 2007, http://www.elme-eu.org/ELME_Results.pdf)
  - A Global Comunity (GC) scenario representing economic growth constrained by common environmental policies reflected by 50 % decrease in riverine nitrate and phosphate (with respect to the present day run) again following the ELME scenario and based on the IPSL A1B simulation.

The *in situ* data used in this study comes from the International Council for the Exploration of the Sea (ICES) EcoSystemData Online Warehouse. Temperature, salinity, nitrate, chlorophyll-a and oxygen data were collected for the top 100m layer. For each *in situ* data record the corresponding (in space and time) model estimate was extracted from the daily-averaged outputs following the procedure established by de Mora et al. (2012).

### Frequency distributions

**Fig. 1:**
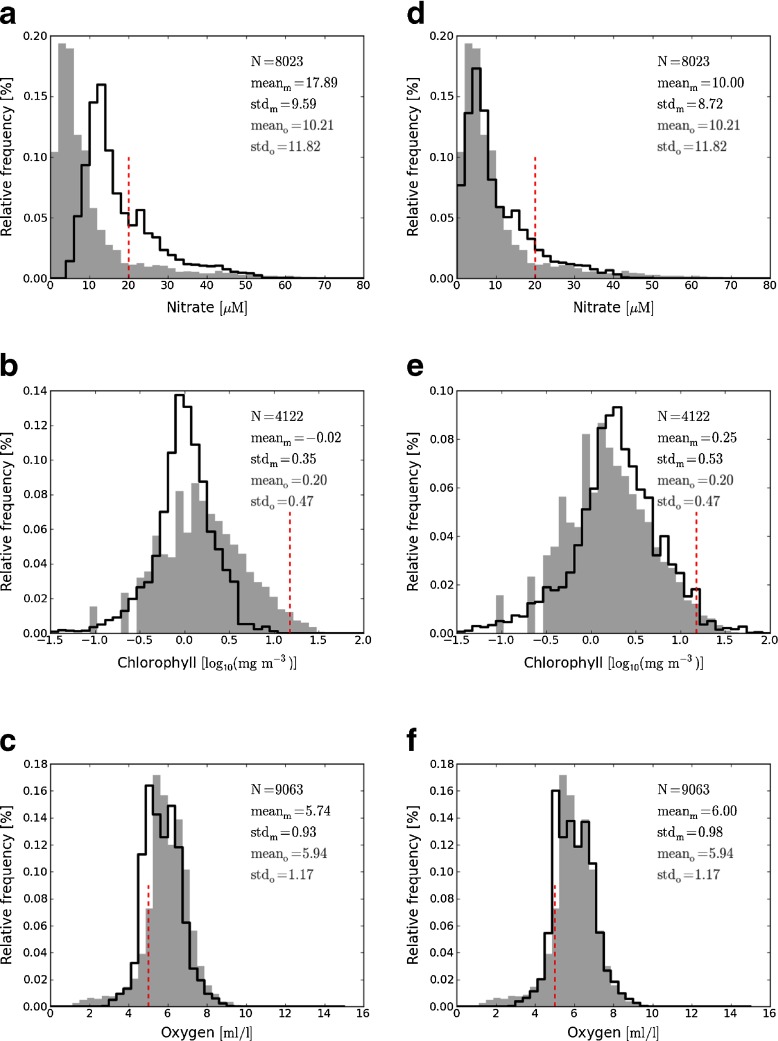
Histogram distribution of model hindcast outputs (*thick black line*) and *in situ* (*grey bars*) data for nitrate concentration, chlorophyll concentration and oxygen concentration (from top to bottom). Left plots (**a,b** and **c**): non-transformed model histograms; right plots (**d**, **e** and **f**): transformed model histograms. Data from 1965 to 1990 for coastal area (salinity between 30 and 34.5) are used. The dashed red line represent the threshold used to determine the risk of eutrophication

Figure 1a, b and c show the model and observation data distributions for the three variables of interest (nitrate (a), chlorophyll (b) and oxygen concentrations (c)) for the hindcast run (1965-1990) and for the time of year as described in Section 2.1.

The number of observations matched up to model output (N) is indicated on each plot and always exceeds 4000. The mean and standard deviation are also shown for each distribution as an indicator of the likeness between model and observed distributions.

For all variables, the distributions of both model and observations have similar shapes, but exhibit some discrepancies. For nitrate, the model distribution is clearly shifted towards high values with a much higher mean value. Indeed, the model distinguishes only two species of inorganic nitrogen (largely corresponding to ammonium and nitrate) but includes other species, like nitrite, not considered in this study. In the case of chlorophyll concentration the model fails to reproduce the right tail of the observational distribution. Oxygen observed distribution is very well reproduced by the model, only a slight negative bias is observed.

These discrepancies, along with previously published comparisons between model and satellite chlorophyll concentrations suggest that the model is not always able to reproduce the absolute values and/or the spatial/temporal patterns (Shutler et al. 2011; Saux Picart et al. 2012). However the similarities in the shape of the distributions suggest that, the model may show some skill when looking at the probability of negative events (exceedance of threshold).

The vertical dashed lines on the plots of Fig. 1 represent the chosen thresholds to determine the risk of eutrophication. Comparing the probability of exceedance of these thresholds, we would get very different results from the model and from observations: consequently, model outputs cannot be used directly to assess the eutrophication risk. Our goal is to be able to realistically estimate the probability of exceedance of a threshold using model outputs. Our approach is to define a transfer functions to correct the model distribution from its bias and spread in order to apply eutrophication status thresholds.

### Model distribution transfer function

In this section we describe the transfer methodology we apply to the model to account for errors in the model simulations.

The transfer function is a simple linear^1^ transformation:

1 $$ M_{\text{transformed}} = \alpha M + \beta $$

where *M* is the model data, *M*_transformed_ is the model data after transformation, and *α* and *β* are two parameters that depend upon characteristics of the model outputs and observation distributions. A quadratic transfer function has also been tested, but did not provide better results.

**Fig. 2:**
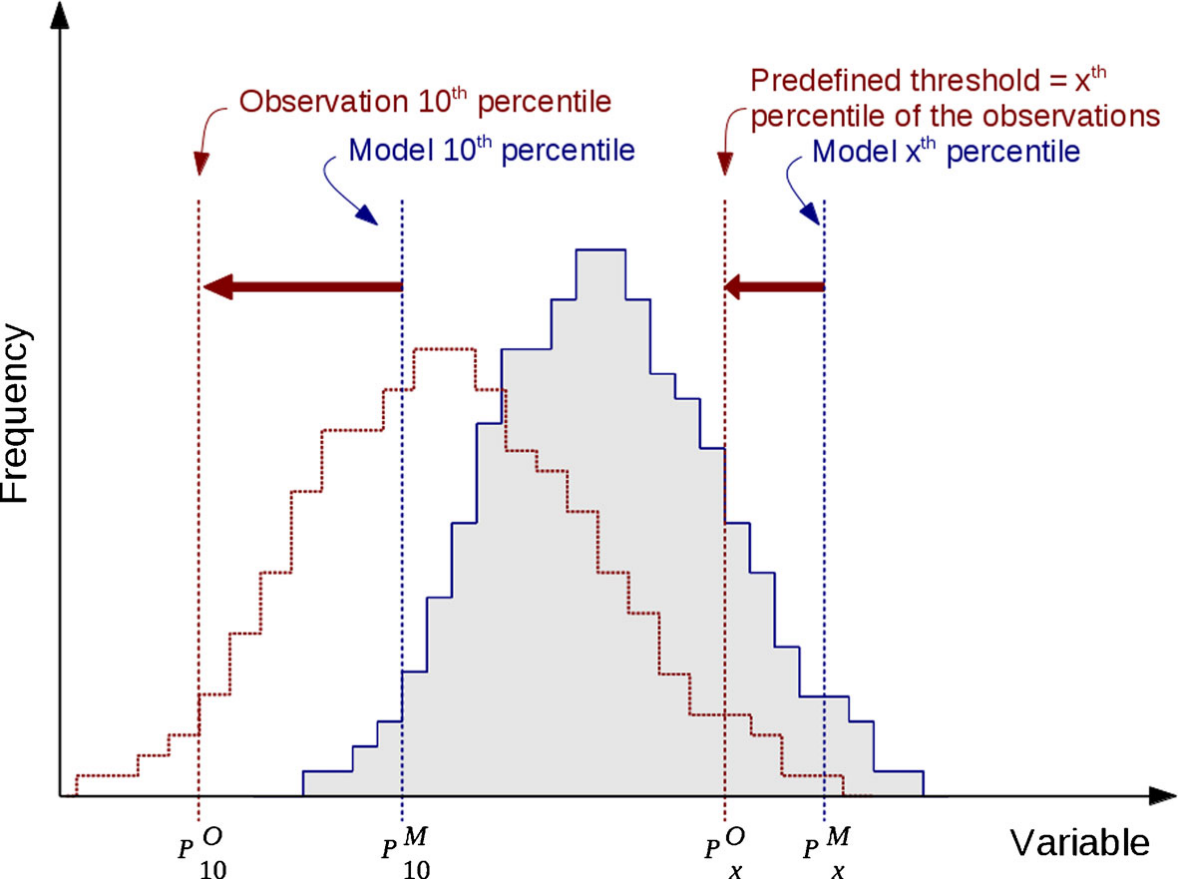
Schematic representation of the transformation applied to P model data. In blue, histogram of the model data; in red dotted line, the transformed histogram

Figure 2 is a schematic illustration of the transformation we apply to the model output. Ideally we would want the model and the observation to give the same probability of exceedance for a given threshold. Using a training sub-sample of the data (defined on a temporal basis between 1965 and 1990) we define *α* and *β* such that the new model distribution (for the same period) has the same 10*th* and *x* ^*th*^ percentile (respectively noted *P*_10_ and *P*_*x*_) as the observation distribution (the *x* ^*th*^ percentile of the observation distribution being the policy-defined threshold *t*).^2^

2 $$\begin{array}{@{}rcl@{}} \alpha &=& \frac{{P_{x}^{O}} - P_{10}^{O}}{{P_{x}^{M}} - P_{10}^{M}} \notag\\ \beta &=& \frac{P_{10}^{O} {P_{x}^{M}} - P_{10}^{M} {P_{x}^{O}}}{{P_{x}^{M}} - P_{10}^{M}} \end{array} $$

where superscripts ^*O*^ and ^*M*^ refer to observation and model respectively. The application of the transfer functions to the model hindcast outputs (1965–1990) is illustrated by Fig. 1d, e and f.

Statistics of the distributions of the observations and model data before and after transformation are shown on Fig. 1. To a first approximation, the transformation applied to model data corrects the model distribution for bias and spread with respect to observation distributions.

### Percentage/probability of exceedance

As explained in Section 2.4 and illustrated on Fig. 1d, e and f, the parameters of the transfer function have been determined for the hindcast run and are reported in the Table 1. The transfer function is then applied to the remainder of the dataset (1990–2004). Having transformed the model distribution using the methodology described above, we can estimate the percentage/probability of exceedance of the thresholds for each year. The percentage exceedance is simply the proportion of values above the thresholds for the transformed hindcast output of the model. In the case of forecast (as we will see in Section 3.2) it is more appropriate to call it a probability of exceedance, this denomination having a direct and intuitive link to the evaluation of risk.

**Table 1:** Parameters of the transfer function *α* and *β* for the variables of interest and for hindcast run (HC) and the present day climate run (PD). Mean values of the parameters’ distribution are presented in bold, minimum and maximum are presented between brackets

		***α*** [*α*_*min*_, *α*_*max*_]	***β*** [*β*_*min*_, *β*_*max*_]
**HC**	Winter nitrate	**0.91** [0.81, 1.01]	**–6.23** [-7.36, -5.31]
	Spring-Summer chl	**1.52** [1.39, 1.60]	**0.28** [0.23, 0.35]
	Summer oxygen	**0.024** [0.023, 0.025]	**–0.1** [-0.36, 0.18]
**PD**	Winter nitrate	**1.16** [1.08, 1.25]	**–10.60** [-11.74, -9.59]
	Spring-Summer chl	**1.31** [1.24, 1.37]	**0.20** [0.16, 0.24]
	Summer oxygen	**0.015** [0.014, 0.016]	**1.51** [1.25, 1.77]

### Uncertainty assessment

When using the methodology described above it is essential to evaluate the degree of confidence we can have in the results. In order to achieve this, we have used a combination of commonly used techniques.

The frequency distribution of the samples may not correspond to the frequency distribution of the whole population (i.e., whole North Sea coastal waters) due to location/time of sampling. We use the bootstrapping methodology (Sheskin 2007) which purpose is to provide the confidence interval associated to the properties of the distributions: $P_{10}^{O}$, ${P_{x}^{O}}$, $P_{10}^{M}$ and ${P_{x}^{M}}$ from Eq. 2.

The propagation of the confidence intervals through to the percentage/probability of exceedance of a threshold is achieved using a Monte-Carlo approach: The parameters of Eq. 2 are randomly sampled (a thousand times) within the bootstrap distribution (95 % confidence interval), and the percentage/probability of exceedance is computed each time giving a distribution of that quantity.

The confidence intervals on the percentage exceedance only account for issues associated with the sampling and the eventuality of outliers. We do not consider uncertainties coming from the model itself (POLCOMS-ERSEM), such as uncertainties on model structure and parameters, or uncertainties associated with forcing data and boundary conditions.

## Results and discussion

This section explores the use of our methodology to predict the risk of eutrophication in the North Sea associated with three different future climatic and anthropogenic scenarios (as described in Section 2.2).

### Percentage exceedance of thresholds using the model hindcast run

**Fig. 3:**
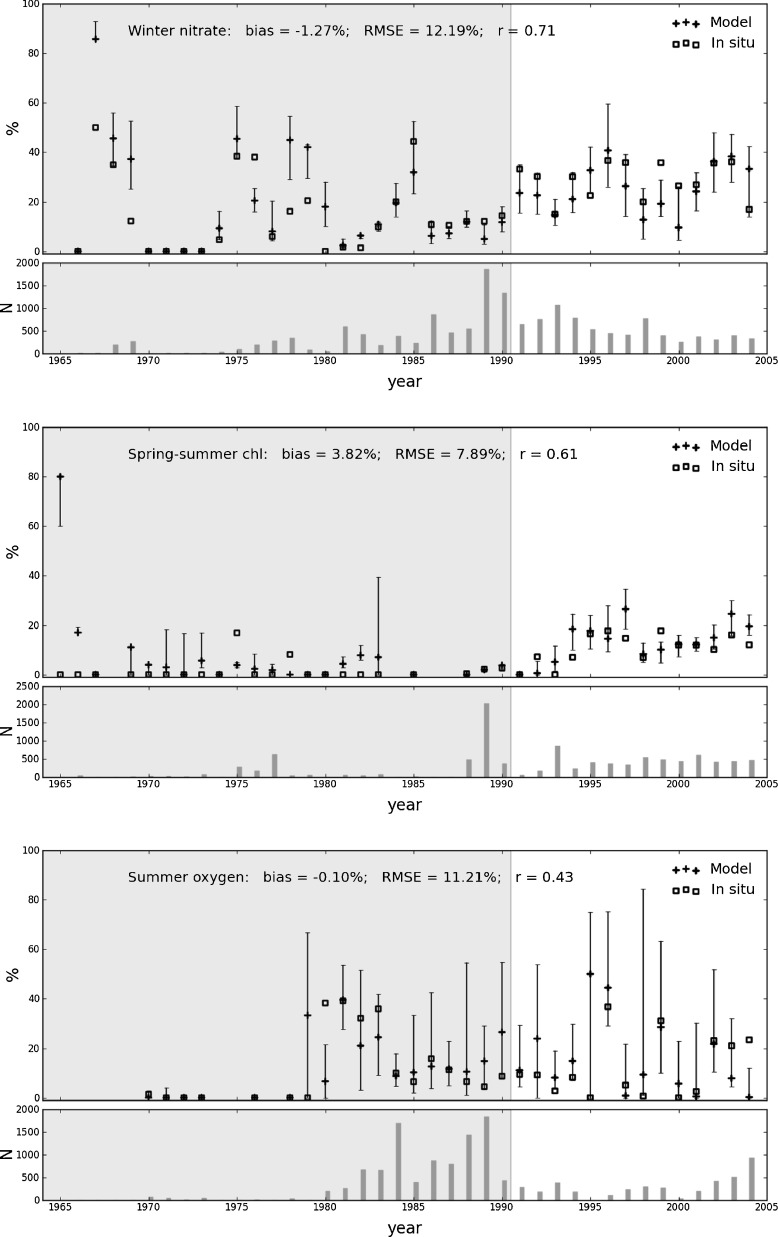
Percentage of exceedance of the thresholds for winter nitrate concentration (*top*), spring-summer chlorophyll concentration (*center*), and summer oxygen (*bottom*) from model hindcast outputs (*crosses* and *error bars*) and observations (*squares*). The bars, in the bottom plot of each pannel, represent the number of observations matched up to model output for each years. The shaded areas correspond to the years used as training data to establish the transfer function. Statistics shown have been estimated using the years for which more than 150 observations were available. All correlations are statistically significant at p-value of 0.05

The transfer function was firstly applied to the matchup database (as described in Section 2.5). Figure 3 shows the percentage of exceedance of the predefined thresholds for both model data (transformed) and observations for winter nitrate, spring-summer chlorophyll and summer oxygen concentrations. It is clear that, after a simple linear transformation, the model is able to predict the percentage of exceedance of thresholds. Most of the cases where the model fails (largely) to predict the percentage of exceedance of thresholds are years for which very few measurements are available (less than one to two hundred data points). The statistics of the comparison between model estimates and observations show that the best results are obtained for the winter nitrate concentration (Fig. 3). However, for all indicators the observed percentage of exceedance consistently falls within the confidence intervals.

For winter nitrate and summer oxygen, the risk of exceeding the threshold varies between 0 % and 40 % while for the spring-summer chlorophyll the risk is below 20 % most of the time. To the first order, the percentage of exceedance is related to the location of the samples. For example the higher percentages observed since 1990 is related to the increasing sampling in “at risk” areas with respect to eutrophication.

**Fig. 4:**
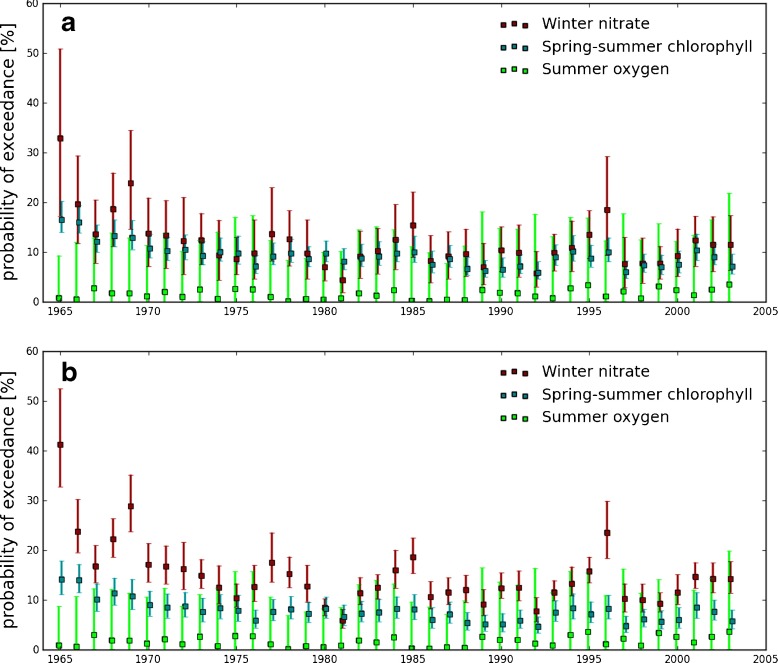
Percentage of exceedance of the thresholds for winter nitrate concentration (*red*), spring-summer chlorophyll concentration (*blue*) and summer oxygen concentration (*green*) from the whole coastal domain of the model hindcast outputs. **a** Training period: 1965–1990, **b** Training period: 1981-2002

Secondly, the transfer function was applied to the whole North Sea coastal domain (as opposed to matchup points only) of the model outputs (Fig. 4a) to assess the risk across the whole region. Winter nitrate and spring-summer chlorophyll concentration both indicate a risk of eutrophication of around 10 % with confidence interval of ±5 to 10 % for the North Sea coastal waters (Fig. 4a). This level is quite constant throughout the years with little inter-annual variations (often co-occuring in both indicators). The risk given by the summer oxygen indicator is much lower (between 0 and 5 %) with larger confidence intervals (10 to 15 %).

### Prediction of eutrophication risk

**Fig. 5:**
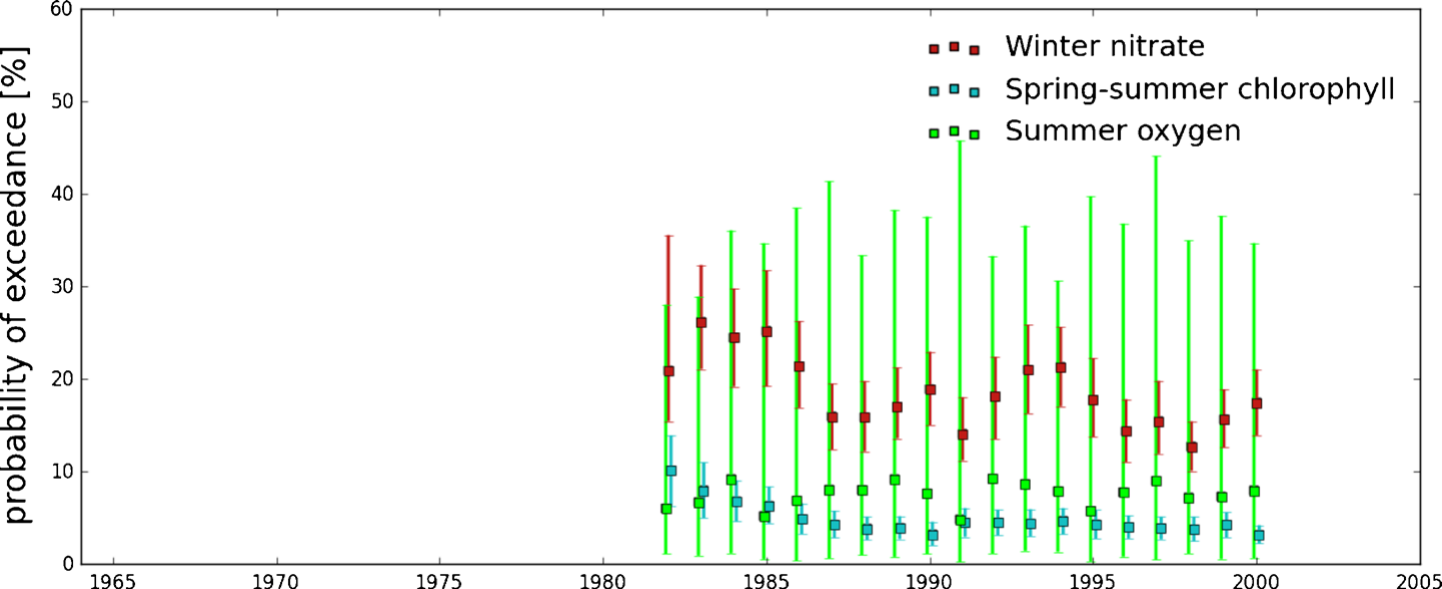
Percentage of exceedance of the thresholds for winter nitrate concentration (*red*), spring-summer chlorophyll concentration (*blue*) and summer oxygen concentration (*green*) from the whole coastal domain of the model present day scenario (PD run) outputs

In this section, we explore the potential of the methodology to project the eutrophication risk under climate change and/or anthropogenic effects. Because the climate forced runs are produced using a different meteorological forcing, we need to redefine the parameters of the transfer function. The matchup between the PD run and *in situ* data (over the years 1981–2002) was performed to determine the parameters of the transfer function and associated confidence intervals (results are reported in Table 1 bottom part). This transfer function was then applied to the North Sea coastal waters of the PD run to estimate the percentage of exceedance of the thresholds for each variable (Fig. 5). In order to compare the percentage/probability exceedance for the hindcast run and the PD run, it was also necessary to redefine the transfer function for the hindcast run using the same training dataset as the one used for the PD run. Figure 4b shows the percentage exceedance of the thresholds for the hindcast run using the 1981–2002 training dataset. We note some differences between Fig. 4a and b. In particular for winter nitrate indicator, for which we observe an increase of almost 3 % in average. Changing the training dataset affect the data distribution and therefore the parameters of the transfer function. This test highlights the limitation of the methodology and the necessity to have a large dataset of observations.

The levels of risk obtained from the hindcast run (Fig. 4b) and the PD run (Fig. 5) show some obvious discrepancies. Firstly, the percentage exceedance for nitrate and oxygen, calculated from the PD run, are higher than in the hindcast, indicating that the forcing plays an important role. Indeed, the forcing data used for the hindcast are expected to include more variability than the IPSL CM data. Secondly, there are also very large error bars on summer oxygen indicator (twice as large as for the hindcast run). This is explained by the fact that the variability of oxygen concentration in the PD run is much lower than that of the hindcast run especially for low concentrations (data not shown). Consequently, the frequency distribution is narrower and has less of a left tail making the detection of the percentage exceedance less accurate.

**Fig. 6:**
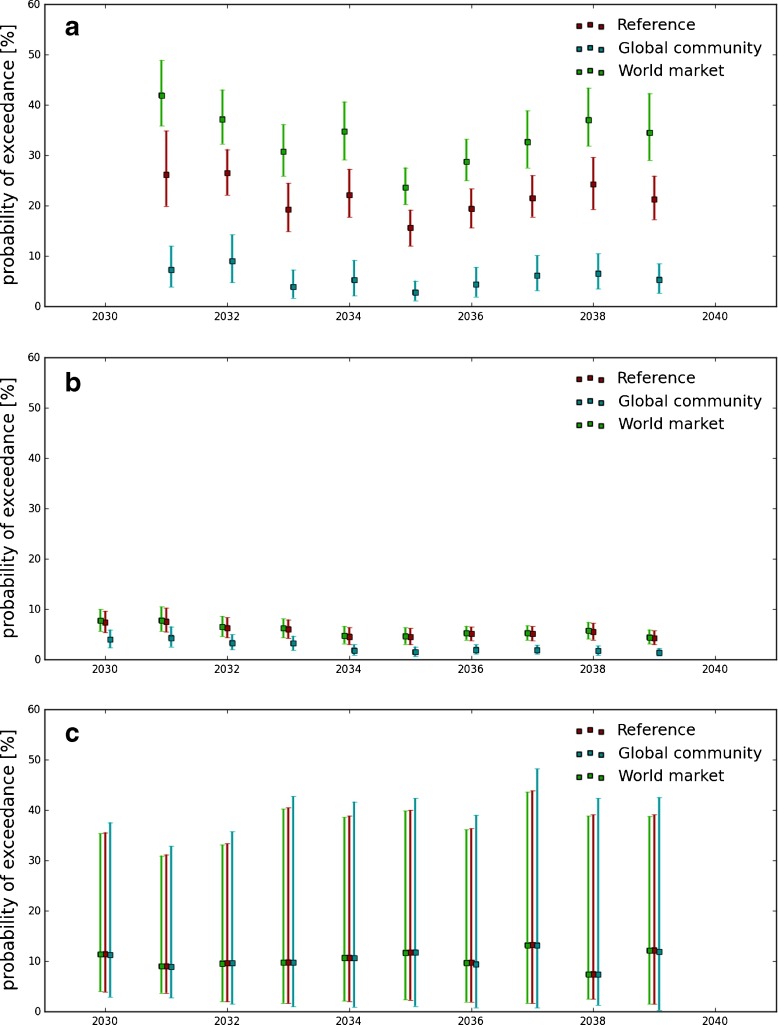
Probability of exceedance of the thresholds given by three different future scenarios for 2030-2040 time period for winter nitrate concentration (**a**), spring-summer chlorophyll concentration (**b**) and summer oxygen concentration (**c**) from the whole coastal domain of the model forecast outputs (reference run in red; Global Community run in blue; World Market run in green)

Figure 6 shows the application of the same transfer function to the three forecast scenarios (reference, GC and WM, see Section 2.2). The reference run and the PD run give similar probability of exceedance (compare Figs. 6 and 5), suggesting that the eutrophication risk may not be sensitive to the climate change signal out to 2040. At this time scale, natural variability probably has a higher impact on eutrophication than climate change and input of nutrient from the rivers.

The nitrate indicator (Fig. 6a) is, as expected, sensitive to nutrient input. Increasing nitrogen input is resulting in an increase in the risk of negative indicator event associated with the nitrate indicator (WM scenario). In the case of chlorophyll (Fig. 6b), it is interesting to note that a decrease in nitrogen and phosphorus input is generating a decrease in the number of negative indicator event, but an increase in nitrogen does not affect the probability of exceedance with respect the reference run. This suggests that nitrogen is not limiting the growth of phytoplankton, instead light, phosphorus and/or grazing may be.

The impact of eutrophication is to adversely drive the planktonic ecosystem. The controls on the planktonic ecosystem of the North Sea include light and nutrient availability, grazing pressure and existing stocks. Cloern (1999) has shown that plankton growth in coastal regions may be light-limited rather than nutrient-limited, McQuatters-Gollop et al. (2007) have shown that this is the case for the North Sea. This behaviour is illustrated by the WM scenario which shows an increase in eutrophication risk in terms of the nitrate indicator but not the chlorophyll indicator. In contrast the GC scenario by significantly reducing the nutrient loads shifts the system from being light limited to nutrient limited as evidenced by the decrease in both the nitrate and chlorophyll indicators. As far as oxygen is concerned, no significant differences are observed between the runs. In well mixed coastal waters, oxygen can rapidly equilibrate with the atmosphere.

Policies addressing eutrophication have been implemented across the North Sea, based on the MSFD and the Water framework directive. The period of the hindcast (1960–2005) covers the period when the eutrophication was at its worst (1980’s) and the more recent efforts to abate land derived nutrient inputs into the North Sea. There are three key aspects which will influence policies at regional level (McQuatters-Gollop et al. 2009); 1) the historic severity of the eutrophication, 2) evidence of recovery and 3) other sources of pressures (e.g., climate change). The results presented demonstrate points 1 and 2, indicating a slow reduction in the eutrophication risk in the 1960’s/70’s and then a stabilisation, subject to interannual variability (Fig. 4). For the third aspect, we should note that eutrophication is only one of the pressures acting on the system and there are confounding effects such as climate and fisheries. The challenge is to tease out in time and space the relative impacts of these pressures. Our results suggest that the risk of eutrophic event is not sensitive to climate change at the timescale in question (+30 years), but is sensitive to anthropogenic impact, in particular land use and agricultural practices (Figs. 5 and 6), implying in the near future, good environemantal status may be compromised by pressures to expand agriculture for food, fibre and biofuels.

### Implications of uncertainty assessment

The methodology presented enables the correction of bias and spread in the distributions of modelled variables with respect to the observational distributions. Confidence intervals have been calculated on the basis of statistical properties (Section 2.6) of the observational frequency distribution. This methodology primarily gives the uncertainty in estimating the transfer function associated to the sampling of the observations. Therefore, it reflects the confidence one can have in the goodness of the transfer function to match the model output distribution to the real distribution of the variable.

For nitrate and chlorophyll indicators, the associated confidence intervals (of the order of 10 %) suggest that the model used can provide reliable estimation of the risk of eutrophication. However the oxygen indicator may not be used as reliably due to very large associated uncertainties. This highlights the major drawback of this methodology, the need for a large database of observations to use as a training database for defining the transfer function. In order to reduce the uncertainties associated to such a definition, the database should be as representative as possible of the real conditions of the ecosystem studied.

Intrinsic uncertainties of the model setup (uncertainties on model parameters, initial conditions or structure of the model) are not taken into consideration in this methodology. However, having defined the methodology, a multi-model or ensemble run would permit the evaluation of those types of uncertainty. Until recently, this has been limited by two factors. The first was defining robust methodologies for the regional downscaling of climate model outputs to drive coupled hydrodynamic ecosystem models with climate model outputs, this has recently been addressed (Holt et al. 2012). The other is computational cost, coupled hydrodynamic ecosystem models are computationally expensive, but the ability to run relatively large ensembles is beginning to be tractable. To further explore and quantify model skill would require a combination of perturbed parameters (e.g., focusing on phytoplankton growth and nutrient recycling parameters) and external forcing (meteorology, ocean boundary conditions, riverine nutrients).

## Conclusion

In this paper, a methodology is proposed to use model outputs to study the risk of eutrophication in the North Sea coastal waters. Despite the discrepancies in modelled and observed frequency distributions, a simple linear transformation enables the use of model outputs for computing percentage/probability of exceedance of thresholds.

Our methodology is generic and has been developed and applied to the whole North Sea coastal domain. However, it may be more suitable, in a policy context, to refine its application to more local areas such as the ones defined in the OSPAR Common Procedure.

This methodology opens up the model tools to new applications in operational oceanography, marine assessment (including management strategy evaluations). It also allows prediction of the risk of eutrophication using scenarios of the future state of the ecosystem, and permits the exploration of the sensitivity of the ecosystem to combinations of climate and management strategies.

Uncertainty assessment has been limited to the distribution of the observations and the definition of the transfer function. Ensemble or multi-models runs would provide estimate of the uncertainty associated with the model(s) and set-up.

The application of this methodology to past and future scenarios may be beneficial to policy development and ecosystem-based management.

## Footnotes

1 The addition of a quadratic term has also been tested, but did not prove to be useful

2 In the case of oxygen, a negative event occures when oxygen concentration is below a minimum threshold, *α* and *β* are therefore defined so that the model and observation distributions have the same *P*_*x*_ and *P*_90_.
